# Poly[[bis­(μ-4,4′-bipyridine-κ^2^ *N*:*N*′)copper(I)] perchlorate 0.24-hydrate]

**DOI:** 10.1107/S1600536812017266

**Published:** 2012-04-25

**Authors:** Chun-Yan Zhang, Xue-Jun Yao, Run-Ling Wang, Cheng-Zhi Xie

**Affiliations:** a Tianjin Key Laboratory on Technologies Enabling Development of Clinical Therapeutics and Diagnostics (Theranostics), School of Pharmacy, Tianjin Medical University, Tianjin 300070, People’s Republic of China; b Clinical Medical College of Tianjin Medical University, Tianjin Medical University, Tianjin 300070, People’s Republic of China; c Department of Pharmacy, The Second Affiliated Hospital of Tianjin Medical University, Tianjin 300211, People’s Republic of China

## Abstract

The title copper(I) polymeric compound, {[Cu(C_10_H_8_N_2_)_2_]ClO_4_·0.24H_2_O}_*n*_, obtained by the reaction of Cu(ClO_4_)_2_ and 4,4′-bipyridine (4,4′-bpy) under hydro­thermal conditions, features a fourfold-inter­penetrated diamondoid coordination framework. The asymmetric unit consists of two Cu^I^ atoms, three 4,4′-bpy ligands in general positions and two halves of two centrosymmetric 4,4′-bpy ligands, two ClO_4_^−^ anions and water mol­ecule with a site-occupancy factor of 0.480 (17). The Cu^I^ atoms are in a distorted tetra­hedral coordination environment and are bridged by 4,4′-bpy ligands, forming a diamondoid cationic polymeric framework that encloses two symmetry-independent channels along [100], which accommodate perchlorate anions and water mol­ecules.

## Related literature

For the use of the 4,4′-bipyridine ligand in the construction of metal-organic frameworks, see: Yaghi & Li (1996 ▶); MacGillivray *et al.* (1994 ▶); Xie *et al.* (2010 ▶). For reduction of Cu^II^ to Cu^I^ and other phenomena occuring under hydro­thermal conditions, see: Liu *et al.* (2001 ▶); Yang *et al.* (2010 ▶); Xie *et al.* (2006 ▶, 2008 ▶). For related structures, see: Pedireddi *et al.* (2006 ▶); Zhang *et al.* (2007 ▶); Qin *et al.* (2007 ▶).
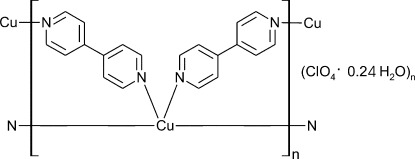


## Experimental

### 

#### Crystal data

[Cu(C_10_H_8_N_2_)_2_]ClO_4_·0.24H_2_O
*M* *_r_* = 479.68
Monoclinic, 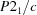

*a* = 7.1894 (14) Å
*b* = 32.380 (7) Å
*c* = 17.319 (4) Å
β = 100.40 (3)°
*V* = 3965.6 (14) Å^3^
*Z* = 8
Mo *K*α radiation
μ = 1.27 mm^−1^
*T* = 293 K
0.26 × 0.11 × 0.11 mm

#### Data collection

Rigaku R-AXIS RAPID IP area-detector diffractometer
Absorption correction: multi-scan (*ABSCOR*; Higashi, 1995 ▶) *T*_min_ = 0.732, *T*_max_ = 0.877
29543 measured reflections
6849 independent reflections
4507 reflections with *I* > 2σ(*I*)
*R*_int_ = 0.095

#### Refinement

*R*[*F* ^2^ > 2σ(*F* ^2^)] = 0.070
*wR*(*F* ^2^) = 0.201
*S* = 1.04
6849 reflections
547 parameters
H-atom parameters constrained
Δρ_max_ = 1.43 e Å^−3^
Δρ_min_ = −0.86 e Å^−3^

### 

Data collection: *RAPID-AUTO* (Rigaku, 2004 ▶); cell refinement: *RAPID-AUTO*; data reduction: *RAPID-AUTO*; program(s) used to solve structure: *SHELXS97* (Sheldrick, 2008 ▶); program(s) used to refine structure: *SHELXL97* (Sheldrick, 2008 ▶); molecular graphics: *SHELXTL* (Sheldrick, 2008 ▶) and *DIAMOND* (Brandenburg & Berndt, 2005 ▶); software used to prepare material for publication: *SHELXTL*.

## Supplementary Material

Crystal structure: contains datablock(s) global, I. DOI: 10.1107/S1600536812017266/gk2464sup1.cif

Structure factors: contains datablock(s) I. DOI: 10.1107/S1600536812017266/gk2464Isup2.hkl

Additional supplementary materials:  crystallographic information; 3D view; checkCIF report

## Figures and Tables

**Table 1:** Selected bond lengths (Å)

N1—Cu1	1.989 (4)
N2—Cu2	2.000 (4)
N3—Cu1	2.078 (5)
N4—Cu2^i^	2.040 (5)
N5—Cu1	2.086 (5)
N6—Cu2^ii^	2.064 (5)
N7—Cu1	2.023 (5)
N8—Cu2	2.057 (5)

Symmetry codes: (i) 
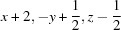
; (ii) 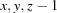.

**Fig. 1.**
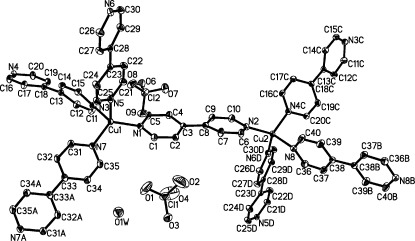
ORTEP presentation of the title compound with 30% probability displacement ellipsoids. All hydrogen atoms are omitted for clarity. Symmetry operations used to generate equivalent atoms: #A -x+1, -y, -z+1; #B -x-3, -y, -z+2; #C x-2, -y+1/2, z+1/2; #D x, y, z+1.

**Fig. 2.**
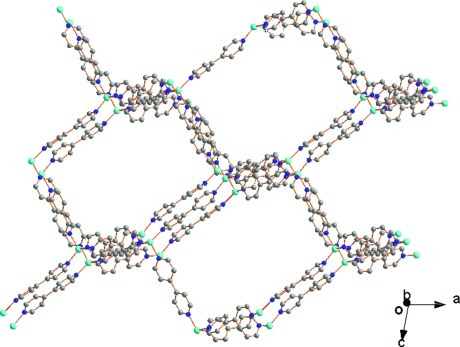
A view of a single {[Cu(C10H8N2)2]+n diamondoid framework in the title compound. Perchlorate anions, water molecules and hydrogen atoms are not shown.

**Fig. 3.**
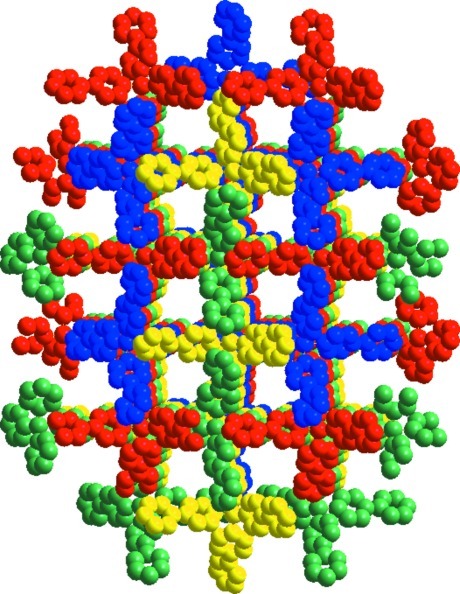
Four-fold interpenetration of the diamondoid frameworks in the title compound viewed along [1 0 0]. Each framework is shown in different colour. Perchlorate anions, water molecules and hydrogen atoms are not shown.

## supplementary crystallographic information

### Comment

The design and synthesis of metal-organic frameworks (MOF) is becoming an increasingly popular field of research in view of the formation of fascinating structures and their potentially useful ion-exchange, adsorption, catalytic, fluorescence and magnetic properties. The simple, rigid, rod-like ligand, 4,4'-bipy, has been used extensively to ligate metal ions into open frameworks with channels (Yaghi & Li, 1996; MacGillivray *et al.*, 1994; Xie *et al.*, 2010). On the other hand, it has been found that under hydrothermal conditions used for preparation of MOFs many interesting phenomena including ligand oxidative coupling, hydrolysis, substitution and redox processes can occur (Liu *et al.*, 2001; Xie *et al.*, 2008). Encouraged by several recent reports on reduction of Cu^II^ to Cu^I^ under basic hydrothermal conditions (Yang *et al.*, 2010; Xie *et al.*, 2006), we designed and synthesized the title three-dimensional copper(I) coordination polymer and determined its crystal structure.

The two symmetry independent Cu^I ^ions are in a distorted tetrahedral coordination geometry, each surrounded by four nitrogen donors from adjacent 4,4'-bpy ligands (Fig. 1). The 4,4'-bpy ligands bridge the Cu^I^ ions into a three dimensional diamodoid framework (Fig. 2) and the crystal structure features a four-fold interpenatration of these frameworks (Fig. 3) leaving two symmetry independent channels along [1 0 0]. These channels are filled with perchlorate counteranions and water molecules. There are three similar examples of diamondoid coordination polymers formed from copper(I) and 4,4'-bipy ligand: [Cu(4,4'-bpy)_2_] NO_3_ (Pedireddi *et al.*, 2006), [Cu_2_ (4,4'-bpy) _4_] (d-Hcam) _2_^.^(4,4'-bipy) _2_^.^12H_2_O(Zhang *et al.*, 2007) and [Cu(4,4'-bpy)_ 2_]ClO_4_ (Qin *et al.*, 2007), which contain similar diamondoid framework but differ in number of interpenetrating networks and crystal symmetry.

### Experimental

A mixture of Cu(ClO_4_)_2_^.^ 6H_2_O (0.186 g, 0.5 mmol), 4,4'-bipy (0.192 g, 1 mmol) and H_2_O (18.0 ml) in the molar ratio of 1:2:1000 was sealed in a 25 mL stainless steel reactor with Teflon liner, and heated directly to 180°C. After keeping at 180°C for 72 h it was cooled slowly to 30°C at a rate of 2°C/h. The resulting orange block crystals were washed and dried in air (yield: 15%.).

### Refinement

The H atoms of the aromatic rings were placed at calculated positions, with C—H = 0.93 Å and assigned *U*_iso_(H) = 1.2Ueq(C). A high peak in a difference Fourier map was interpreted as a water molecule with partial occupancy. The occupancy factor of water molecule refined at 0.480 (17). Hydrogen atoms of water molecule could not be located. O1W was refined with isotropic displacement parameter.

### Crystal data

**Table:**

[Cu(C_10_H_8_N_2_)_2_]ClO_4_·0.24H_2_O	*F*(000) = 1955
*M**_r_* = 479.68	*D*_x_ = 1.607 Mg m^−^^3^
Monoclinic, *P*2_1_/*c*	Mo *K*α radiation, λ = 0.71073 Å
Hall symbol: -P 2ybc	Cell parameters from 36980 reflections
*a* = 7.1894 (14) Å	θ = 3.1–25.5°
*b* = 32.380 (7) Å	µ = 1.27 mm^−^^1^
*c* = 17.319 (4) Å	*T* = 293 K
β = 100.40 (3)°	Block, orange
*V* = 3965.6 (14) Å^3^	0.26 × 0.11 × 0.11 mm
*Z* = 8	

### Data collection

**Table:**

Rigaku R-AXIS RAPID IP area-detector diffractometer	6849 independent reflections
Radiation source: fine-focus sealed tube	4507 reflections with *I* > 2σ(*I*)
Graphite monochromator	*R*_int_ = 0.095
ω scans	θ_max_ = 25.0°, θ_min_ = 3.1°
Absorption correction: multi-scan (*ABSCOR*; Higashi, 1995)	*h* = −8→8
*T*_min_ = 0.732, *T*_max_ = 0.877	*k* = −38→38
29543 measured reflections	*l* = −20→20

### Refinement

**Table:**

Refinement on *F*^2^	Primary atom site location: structure-invariant direct methods
Least-squares matrix: full	Secondary atom site location: difference Fourier map
*R*[*F*^2^ > 2σ(*F*^2^)] = 0.070	Hydrogen site location: inferred from neighbouring sites
*wR*(*F*^2^) = 0.201	H-atom parameters constrained
*S* = 1.04	*w* = 1/[σ^2^(*F*_o_^2^) + (0.0887*P*)^2^ + 9.2459*P*] where *P* = (*F*_o_^2^ + 2*F*_c_^2^)/3
6849 reflections	(Δ/σ)_max_ < 0.001
547 parameters	Δρ_max_ = 1.43 e Å^−^^3^
0 restraints	Δρ_min_ = −0.86 e Å^−^^3^

### Special details

**Table:**

Geometry. All esds (except the esd in the dihedral angle between two l.s. planes) are estimated using the full covariance matrix. The cell esds are taken into account individually in the estimation of esds in distances, angles and torsion angles; correlations between esds in cell parameters are only used when they are defined by crystal symmetry. An approximate (isotropic) treatment of cell esds is used for estimating esds involving l.s. planes.
Refinement. Refinement of *F*^2^ against ALL reflections. The weighted *R*-factor *wR* and goodness of fit *S* are based on *F*^2^, conventional *R*-factors *R* are based on *F*, with *F* set to zero for negative *F*^2^. The threshold expression of *F*^2^ > σ(*F*^2^) is used only for calculating *R*-factors(gt) *etc*. and is not relevant to the choice of reflections for refinement. *R*-factors based on *F*^2^ are statistically about twice as large as those based on *F*, and *R*- factors based on ALL data will be even larger.

### Fractional atomic coordinates and isotropic or equivalent isotropic displacement parameters (Å^2^)

**Table:**

	*x*	*y*	*z*	*U*_iso_*/*U*_eq_	Occ. (<1)
C1	−0.2429 (8)	0.09009 (17)	0.6490 (4)	0.0320 (14)	
H1	−0.2089	0.0651	0.6289	0.038*	
C2	−0.3824 (8)	0.08945 (17)	0.6942 (3)	0.0307 (13)	
H2	−0.4415	0.0647	0.7026	0.037*	
C3	−0.4345 (8)	0.12579 (17)	0.7270 (3)	0.0272 (12)	
C4	−0.3394 (9)	0.16107 (17)	0.7119 (4)	0.0344 (14)	
H4	−0.3671	0.1862	0.7334	0.041*	
C5	−0.2037 (8)	0.15924 (17)	0.6653 (4)	0.0329 (14)	
H5	−0.1432	0.1836	0.6560	0.039*	
C6	−0.8364 (9)	0.09490 (19)	0.8285 (4)	0.0378 (15)	
H6	−0.9146	0.0722	0.8301	0.045*	
C7	−0.7093 (9)	0.09400 (19)	0.7771 (4)	0.0380 (15)	
H7	−0.7070	0.0715	0.7439	0.046*	
C8	−0.5853 (8)	0.12672 (16)	0.7754 (3)	0.0274 (12)	
C9	−0.6128 (9)	0.16069 (18)	0.8199 (3)	0.0343 (14)	
H9	−0.5412	0.1844	0.8171	0.041*	
C10	−0.7446 (9)	0.16006 (18)	0.8682 (4)	0.0360 (15)	
H10	−0.7601	0.1837	0.8969	0.043*	
C11	0.2471 (9)	0.20186 (18)	0.6009 (3)	0.0335 (14)	
H11	0.2313	0.1948	0.6514	0.040*	
C12	0.3796 (9)	0.23233 (19)	0.5929 (3)	0.0348 (14)	
H12	0.4467	0.2456	0.6368	0.042*	
C13	0.4093 (8)	0.24236 (16)	0.5185 (3)	0.0280 (13)	
C14	0.2909 (8)	0.22430 (17)	0.4555 (3)	0.0310 (13)	
H14	0.2997	0.2319	0.4045	0.037*	
C15	0.1605 (9)	0.19513 (17)	0.4680 (3)	0.0304 (13)	
H15	0.0818	0.1838	0.4247	0.036*	
C16	0.7924 (9)	0.29751 (18)	0.4372 (4)	0.0352 (14)	
H16	0.8650	0.2931	0.3986	0.042*	
C17	0.6609 (9)	0.26805 (19)	0.4472 (3)	0.0355 (14)	
H17	0.6426	0.2451	0.4145	0.043*	
C18	0.5554 (8)	0.27271 (17)	0.5065 (3)	0.0278 (12)	
C19	0.5869 (9)	0.30835 (18)	0.5514 (4)	0.0343 (14)	
H19	0.5204	0.3129	0.5919	0.041*	
C20	0.7157 (9)	0.33673 (19)	0.5360 (4)	0.0366 (15)	
H20	0.7317	0.3607	0.5660	0.044*	
C21	−0.3331 (9)	0.14752 (18)	0.4278 (3)	0.0342 (14)	
H21	−0.3599	0.1640	0.4685	0.041*	
C22	−0.4564 (9)	0.14808 (19)	0.3586 (3)	0.0352 (14)	
H22	−0.5655	0.1640	0.3533	0.042*	
C23	−0.4197 (8)	0.12482 (16)	0.2958 (3)	0.0261 (12)	
C24	−0.2568 (9)	0.1018 (2)	0.3082 (4)	0.0414 (16)	
H24	−0.2257	0.0860	0.2675	0.050*	
C25	−0.1385 (9)	0.1020 (2)	0.3803 (4)	0.0386 (15)	
H25	−0.0298	0.0858	0.3872	0.046*	
C26	−0.6627 (9)	0.09736 (18)	0.0896 (4)	0.0342 (14)	
H26	−0.6517	0.0774	0.0521	0.041*	
C27	−0.5376 (8)	0.09632 (18)	0.1602 (3)	0.0326 (13)	
H27	−0.4438	0.0762	0.1690	0.039*	
C28	−0.5519 (8)	0.12528 (16)	0.2182 (3)	0.0267 (12)	
C29	−0.6942 (8)	0.15461 (19)	0.1994 (3)	0.0341 (14)	
H29	−0.7087	0.1751	0.2355	0.041*	
C30	−0.8129 (8)	0.15356 (18)	0.1282 (3)	0.0329 (14)	
H30	−0.9084	0.1732	0.1179	0.040*	
C31	0.3277 (10)	0.0863 (2)	0.4967 (4)	0.0475 (19)	
H31	0.3389	0.1124	0.4758	0.057*	
C32	0.4468 (9)	0.05619 (19)	0.4798 (4)	0.0428 (17)	
H32	0.5359	0.0624	0.4487	0.051*	
C33	0.4365 (8)	0.01689 (18)	0.5083 (3)	0.0302 (13)	
C34	0.3011 (11)	0.0112 (2)	0.5549 (5)	0.058 (2)	
H34	0.2883	−0.0145	0.5771	0.070*	
C35	0.1855 (10)	0.0430 (2)	0.5688 (5)	0.051 (2)	
H35	0.0945	0.0376	0.5994	0.061*	
C36	−1.1328 (9)	0.0501 (2)	1.0417 (4)	0.0392 (15)	
H36	−1.0340	0.0554	1.0832	0.047*	
C37	−1.2718 (9)	0.02264 (19)	1.0534 (4)	0.0367 (14)	
H37	−1.2641	0.0098	1.1020	0.044*	
C38	−1.4211 (8)	0.01396 (17)	0.9944 (3)	0.0287 (12)	
C39	−1.4179 (10)	0.0331 (2)	0.9230 (4)	0.0467 (17)	
H39	−1.5127	0.0277	0.8800	0.056*	
C40	−1.2740 (10)	0.0599 (2)	0.9161 (4)	0.0480 (18)	
H40	−1.2755	0.0722	0.8674	0.058*	
N1	−0.1530 (7)	0.12431 (13)	0.6323 (3)	0.0273 (10)	
N2	−0.8522 (7)	0.12694 (14)	0.8760 (3)	0.0279 (10)	
N3	0.1415 (7)	0.18220 (14)	0.5406 (3)	0.0321 (11)	
N4	0.8213 (7)	0.33187 (15)	0.4796 (3)	0.0313 (11)	
N5	−0.1744 (7)	0.12460 (13)	0.4413 (3)	0.0270 (10)	
N6	−0.7985 (7)	0.12572 (14)	0.0731 (3)	0.0296 (11)	
N7	0.1957 (7)	0.08080 (15)	0.5413 (3)	0.0299 (11)	
N8	−1.1333 (7)	0.06960 (14)	0.9737 (3)	0.0325 (11)	
O1	−0.0666 (15)	−0.0130 (2)	0.6676 (5)	0.139 (4)	
O2	−0.3401 (12)	−0.0158 (3)	0.7042 (7)	0.164 (4)	
O3	−0.0968 (9)	−0.06143 (15)	0.7597 (4)	0.0711 (18)	
O4	−0.1105 (17)	0.0063 (2)	0.7922 (5)	0.148 (4)	
O6	−0.0371 (9)	0.30827 (15)	0.7768 (3)	0.0673 (17)	
O7	−0.2910 (7)	0.26223 (16)	0.7630 (3)	0.0585 (14)	
O8	−0.0905 (8)	0.26365 (15)	0.6697 (3)	0.0589 (15)	
O9	0.0181 (8)	0.23755 (19)	0.7945 (3)	0.0713 (16)	
O1W	0.3437 (18)	−0.0047 (4)	0.7547 (7)	0.073 (5)*	0.480 (17)
Cl1	−0.1334 (3)	−0.02079 (5)	0.73353 (11)	0.0575 (6)	
Cl2	−0.0999 (2)	0.26851 (5)	0.75064 (9)	0.0403 (4)	
Cu1	0.00680 (10)	0.12611 (2)	0.54984 (4)	0.0274 (2)	
Cu2	−0.98541 (10)	0.12353 (2)	0.96766 (4)	0.0285 (2)	

### Atomic displacement parameters (Å^2^)

**Table:**

	*U*^11^	*U*^22^	*U*^33^	*U*^12^	*U*^13^	*U*^23^
C1	0.035 (3)	0.025 (3)	0.039 (3)	−0.002 (2)	0.014 (3)	−0.003 (2)
C2	0.030 (3)	0.025 (3)	0.039 (3)	−0.002 (2)	0.010 (3)	0.000 (2)
C3	0.024 (3)	0.031 (3)	0.027 (3)	0.000 (2)	0.007 (3)	0.005 (2)
C4	0.040 (4)	0.022 (3)	0.045 (4)	−0.004 (3)	0.019 (3)	−0.003 (2)
C5	0.034 (3)	0.027 (3)	0.042 (3)	−0.008 (3)	0.018 (3)	0.000 (3)
C6	0.034 (3)	0.034 (3)	0.050 (4)	−0.010 (3)	0.019 (3)	−0.004 (3)
C7	0.043 (4)	0.037 (3)	0.040 (4)	−0.009 (3)	0.022 (3)	−0.011 (3)
C8	0.027 (3)	0.025 (3)	0.031 (3)	−0.001 (2)	0.007 (3)	−0.001 (2)
C9	0.039 (4)	0.032 (3)	0.035 (3)	−0.005 (3)	0.014 (3)	0.001 (3)
C10	0.044 (4)	0.027 (3)	0.042 (4)	−0.001 (3)	0.021 (3)	−0.006 (3)
C11	0.041 (4)	0.033 (3)	0.027 (3)	−0.006 (3)	0.009 (3)	0.002 (3)
C12	0.036 (3)	0.042 (4)	0.025 (3)	−0.011 (3)	0.002 (3)	−0.004 (3)
C13	0.026 (3)	0.023 (3)	0.037 (3)	−0.001 (2)	0.011 (3)	0.001 (2)
C14	0.035 (3)	0.031 (3)	0.027 (3)	0.002 (3)	0.005 (3)	0.002 (2)
C15	0.036 (3)	0.027 (3)	0.029 (3)	−0.003 (2)	0.006 (3)	−0.001 (2)
C16	0.039 (4)	0.037 (3)	0.034 (3)	−0.004 (3)	0.017 (3)	−0.008 (3)
C17	0.040 (4)	0.039 (3)	0.031 (3)	−0.011 (3)	0.014 (3)	−0.007 (3)
C18	0.023 (3)	0.031 (3)	0.030 (3)	−0.005 (2)	0.006 (3)	0.001 (2)
C19	0.030 (3)	0.037 (3)	0.039 (3)	−0.001 (3)	0.015 (3)	0.001 (3)
C20	0.035 (3)	0.038 (3)	0.040 (4)	−0.007 (3)	0.015 (3)	−0.012 (3)
C21	0.039 (4)	0.034 (3)	0.030 (3)	0.009 (3)	0.007 (3)	−0.003 (3)
C22	0.036 (3)	0.038 (3)	0.032 (3)	0.011 (3)	0.006 (3)	−0.002 (3)
C23	0.026 (3)	0.022 (3)	0.032 (3)	0.000 (2)	0.010 (3)	0.002 (2)
C24	0.039 (4)	0.044 (4)	0.042 (4)	0.014 (3)	0.012 (3)	−0.012 (3)
C25	0.036 (4)	0.047 (4)	0.033 (3)	0.012 (3)	0.006 (3)	−0.007 (3)
C26	0.036 (3)	0.033 (3)	0.033 (3)	0.005 (3)	0.005 (3)	−0.007 (3)
C27	0.028 (3)	0.035 (3)	0.033 (3)	0.008 (3)	0.001 (3)	−0.002 (3)
C28	0.027 (3)	0.022 (3)	0.034 (3)	−0.004 (2)	0.014 (3)	0.002 (2)
C29	0.033 (3)	0.038 (3)	0.032 (3)	0.006 (3)	0.005 (3)	−0.005 (3)
C30	0.033 (3)	0.031 (3)	0.036 (3)	0.008 (3)	0.009 (3)	−0.003 (3)
C31	0.050 (4)	0.030 (3)	0.072 (5)	0.010 (3)	0.038 (4)	0.013 (3)
C32	0.039 (4)	0.033 (3)	0.065 (4)	0.004 (3)	0.033 (4)	0.008 (3)
C33	0.025 (3)	0.032 (3)	0.034 (3)	−0.002 (2)	0.005 (3)	−0.006 (2)
C34	0.065 (5)	0.033 (4)	0.091 (6)	0.017 (3)	0.052 (5)	0.025 (4)
C35	0.045 (4)	0.042 (4)	0.077 (5)	0.008 (3)	0.039 (4)	0.016 (4)
C36	0.034 (3)	0.051 (4)	0.031 (3)	−0.005 (3)	−0.001 (3)	0.004 (3)
C37	0.039 (4)	0.041 (4)	0.028 (3)	−0.008 (3)	0.002 (3)	0.006 (3)
C38	0.028 (3)	0.029 (3)	0.030 (3)	0.002 (2)	0.006 (3)	0.000 (2)
C39	0.044 (4)	0.062 (5)	0.029 (3)	−0.017 (3)	−0.006 (3)	0.014 (3)
C40	0.046 (4)	0.065 (5)	0.032 (4)	−0.018 (4)	0.003 (3)	0.012 (3)
N1	0.027 (2)	0.027 (2)	0.029 (3)	0.002 (2)	0.006 (2)	−0.0013 (19)
N2	0.023 (2)	0.028 (2)	0.034 (3)	0.000 (2)	0.008 (2)	−0.004 (2)
N3	0.034 (3)	0.027 (3)	0.036 (3)	−0.001 (2)	0.009 (2)	0.001 (2)
N4	0.029 (3)	0.036 (3)	0.031 (3)	−0.006 (2)	0.010 (2)	−0.003 (2)
N5	0.026 (2)	0.025 (2)	0.031 (3)	−0.0022 (19)	0.008 (2)	−0.002 (2)
N6	0.023 (2)	0.031 (3)	0.036 (3)	0.000 (2)	0.009 (2)	−0.003 (2)
N7	0.025 (3)	0.035 (3)	0.030 (3)	0.002 (2)	0.005 (2)	0.001 (2)
N8	0.032 (3)	0.030 (3)	0.036 (3)	−0.001 (2)	0.008 (3)	0.004 (2)
O1	0.239 (11)	0.061 (4)	0.159 (8)	0.025 (5)	0.145 (8)	0.038 (5)
O2	0.081 (6)	0.145 (8)	0.247 (13)	0.027 (5)	−0.019 (7)	0.036 (8)
O3	0.085 (4)	0.047 (3)	0.095 (4)	0.021 (3)	0.053 (4)	0.026 (3)
O4	0.254 (12)	0.083 (5)	0.088 (6)	0.005 (6)	−0.017 (7)	−0.036 (4)
O6	0.091 (4)	0.048 (3)	0.070 (4)	−0.034 (3)	0.034 (4)	−0.021 (3)
O7	0.044 (3)	0.063 (3)	0.072 (4)	−0.012 (2)	0.019 (3)	−0.008 (3)
O8	0.094 (4)	0.051 (3)	0.036 (3)	0.004 (3)	0.024 (3)	0.002 (2)
O9	0.068 (4)	0.082 (4)	0.058 (4)	0.008 (3)	−0.005 (3)	0.027 (3)
Cl1	0.0898 (15)	0.0381 (9)	0.0517 (11)	0.0202 (9)	0.0316 (11)	0.0095 (8)
Cl2	0.0521 (10)	0.0365 (8)	0.0328 (8)	−0.0097 (7)	0.0087 (8)	−0.0008 (6)
Cu1	0.0257 (4)	0.0265 (4)	0.0330 (4)	0.0005 (3)	0.0132 (3)	0.0002 (3)
Cu2	0.0254 (4)	0.0294 (4)	0.0336 (4)	0.0021 (3)	0.0131 (3)	0.0015 (3)

### Geometric parameters (Å, º)

**Table:**

C1—N1	1.340 (7)	C25—N5	1.348 (7)
C1—C2	1.379 (7)	C25—H25	0.9300
C1—H1	0.9300	C26—N6	1.333 (7)
C2—C3	1.388 (7)	C26—C27	1.381 (8)
C2—H2	0.9300	C26—H26	0.9300
C3—C4	1.380 (8)	C27—C28	1.391 (8)
C3—C8	1.486 (8)	C27—H27	0.9300
C4—C5	1.375 (7)	C28—C29	1.391 (8)
C4—H4	0.9300	C29—C30	1.367 (8)
C5—N1	1.347 (7)	C29—H29	0.9300
C5—H5	0.9300	C30—N6	1.331 (7)
C6—N2	1.342 (7)	C30—H30	0.9300
C6—C7	1.385 (8)	C31—N7	1.339 (7)
C6—H6	0.9300	C31—C32	1.364 (8)
C7—C8	1.388 (8)	C31—H31	0.9300
C7—H7	0.9300	C32—C33	1.372 (8)
C8—C9	1.377 (8)	C32—H32	0.9300
C9—C10	1.373 (8)	C33—C34	1.384 (8)
C9—H9	0.9300	C33—C33^i^	1.486 (11)
C10—N2	1.343 (7)	C34—C35	1.370 (9)
C10—H10	0.9300	C34—H34	0.9300
C11—N3	1.336 (8)	C35—N7	1.320 (8)
C11—C12	1.395 (8)	C35—H35	0.9300
C11—H11	0.9300	C36—N8	1.336 (7)
C12—C13	1.383 (8)	C36—C37	1.381 (9)
C12—H12	0.9300	C36—H36	0.9300
C13—C14	1.386 (8)	C37—C38	1.371 (9)
C13—C18	1.480 (8)	C37—H37	0.9300
C14—C15	1.376 (8)	C38—C39	1.388 (8)
C14—H14	0.9300	C38—C38^ii^	1.490 (11)
C15—N3	1.355 (7)	C39—C40	1.373 (9)
C15—H15	0.9300	C39—H39	0.9300
C16—N4	1.328 (7)	C40—N8	1.324 (8)
C16—C17	1.376 (8)	C40—H40	0.9300
C16—H16	0.9300	N1—Cu1	1.989 (4)
C17—C18	1.391 (7)	N2—Cu2	2.000 (4)
C17—H17	0.9300	N3—Cu1	2.078 (5)
C18—C19	1.387 (8)	N4—Cu2^iii^	2.040 (5)
C19—C20	1.364 (8)	N5—Cu1	2.086 (5)
C19—H19	0.9300	N6—Cu2^iv^	2.064 (5)
C20—N4	1.351 (7)	N7—Cu1	2.023 (5)
C20—H20	0.9300	N8—Cu2	2.057 (5)
C21—N5	1.346 (7)	O1—Cl1	1.340 (6)
C21—C22	1.356 (9)	O2—Cl1	1.490 (9)
C21—H21	0.9300	O3—Cl1	1.401 (5)
C22—C23	1.388 (8)	O4—Cl1	1.330 (7)
C22—H22	0.9300	O6—Cl2	1.412 (5)
C23—C24	1.371 (8)	O7—Cl2	1.442 (5)
C23—C28	1.499 (9)	O8—Cl2	1.424 (5)
C24—C25	1.379 (9)	O9—Cl2	1.438 (6)
C24—H24	0.9300		
			
N1—C1—C2	124.3 (5)	C30—C29—C28	120.5 (5)
N1—C1—H1	117.9	C30—C29—H29	119.8
C2—C1—H1	117.9	C28—C29—H29	119.8
C1—C2—C3	119.8 (5)	N6—C30—C29	123.2 (5)
C1—C2—H2	120.1	N6—C30—H30	118.4
C3—C2—H2	120.1	C29—C30—H30	118.4
C4—C3—C2	116.4 (5)	N7—C31—C32	124.6 (6)
C4—C3—C8	121.8 (5)	N7—C31—H31	117.7
C2—C3—C8	121.8 (5)	C32—C31—H31	117.7
C5—C4—C3	120.2 (5)	C31—C32—C33	120.6 (5)
C5—C4—H4	119.9	C31—C32—H32	119.7
C3—C4—H4	119.9	C33—C32—H32	119.7
N1—C5—C4	124.1 (5)	C32—C33—C34	114.9 (5)
N1—C5—H5	118.0	C32—C33—C33^i^	122.5 (6)
C4—C5—H5	118.0	C34—C33—C33^i^	122.5 (7)
N2—C6—C7	123.3 (5)	C35—C34—C33	121.1 (6)
N2—C6—H6	118.4	C35—C34—H34	119.5
C7—C6—H6	118.4	C33—C34—H34	119.5
C6—C7—C8	119.8 (5)	N7—C35—C34	123.9 (6)
C6—C7—H7	120.1	N7—C35—H35	118.1
C8—C7—H7	120.1	C34—C35—H35	118.1
C9—C8—C7	116.1 (5)	N8—C36—C37	123.2 (6)
C9—C8—C3	121.6 (5)	N8—C36—H36	118.4
C7—C8—C3	122.2 (5)	C37—C36—H36	118.4
C10—C9—C8	120.9 (5)	C38—C37—C36	120.9 (6)
C10—C9—H9	119.6	C38—C37—H37	119.6
C8—C9—H9	119.6	C36—C37—H37	119.6
N2—C10—C9	123.1 (5)	C37—C38—C39	115.9 (5)
N2—C10—H10	118.4	C37—C38—C38^ii^	122.7 (6)
C9—C10—H10	118.4	C39—C38—C38^ii^	121.4 (7)
N3—C11—C12	124.1 (5)	C40—C39—C38	119.6 (7)
N3—C11—H11	117.9	C40—C39—H39	120.2
C12—C11—H11	117.9	C38—C39—H39	120.2
C13—C12—C11	118.8 (6)	N8—C40—C39	124.7 (6)
C13—C12—H12	120.6	N8—C40—H40	117.6
C11—C12—H12	120.6	C39—C40—H40	117.6
C12—C13—C14	117.3 (5)	C1—N1—C5	115.1 (5)
C12—C13—C18	121.3 (6)	C1—N1—Cu1	122.9 (4)
C14—C13—C18	121.4 (5)	C5—N1—Cu1	121.0 (4)
C15—C14—C13	120.3 (5)	C6—N2—C10	116.2 (5)
C15—C14—H14	119.9	C6—N2—Cu2	123.4 (4)
C13—C14—H14	119.9	C10—N2—Cu2	119.4 (4)
N3—C15—C14	123.0 (6)	C11—N3—C15	116.1 (5)
N3—C15—H15	118.5	C11—N3—Cu1	124.3 (4)
C14—C15—H15	118.5	C15—N3—Cu1	117.9 (4)
N4—C16—C17	123.6 (5)	C16—N4—C20	116.5 (5)
N4—C16—H16	118.2	C16—N4—Cu2^iii^	125.3 (4)
C17—C16—H16	118.2	C20—N4—Cu2^iii^	118.2 (4)
C16—C17—C18	119.8 (5)	C21—N5—C25	116.1 (5)
C16—C17—H17	120.1	C21—N5—Cu1	121.0 (4)
C18—C17—H17	120.1	C25—N5—Cu1	122.8 (4)
C19—C18—C17	116.6 (5)	C30—N6—C26	117.5 (5)
C19—C18—C13	121.5 (5)	C30—N6—Cu2^iv^	122.4 (4)
C17—C18—C13	121.8 (5)	C26—N6—Cu2^iv^	120.1 (4)
C20—C19—C18	120.0 (5)	C35—N7—C31	115.0 (5)
C20—C19—H19	120.0	C35—N7—Cu1	124.4 (4)
C18—C19—H19	120.0	C31—N7—Cu1	120.1 (4)
N4—C20—C19	123.5 (5)	C40—N8—C36	115.7 (5)
N4—C20—H20	118.2	C40—N8—Cu2	119.6 (4)
C19—C20—H20	118.2	C36—N8—Cu2	121.7 (4)
N5—C21—C22	124.0 (5)	O4—Cl1—O1	120.9 (6)
N5—C21—H21	118.0	O4—Cl1—O3	112.5 (5)
C22—C21—H21	118.0	O1—Cl1—O3	112.1 (4)
C21—C22—C23	120.0 (6)	O4—Cl1—O2	99.6 (7)
C21—C22—H22	120.0	O1—Cl1—O2	100.3 (7)
C23—C22—H22	120.0	O3—Cl1—O2	109.3 (5)
C24—C23—C22	116.7 (6)	O6—Cl2—O8	110.4 (3)
C24—C23—C28	122.1 (5)	O6—Cl2—O9	110.0 (4)
C22—C23—C28	121.2 (5)	O8—Cl2—O9	108.3 (3)
C23—C24—C25	120.6 (5)	O6—Cl2—O7	109.8 (3)
C23—C24—H24	119.7	O8—Cl2—O7	110.2 (4)
C25—C24—H24	119.7	O9—Cl2—O7	108.1 (3)
N5—C25—C24	122.5 (6)	N1—Cu1—N7	120.93 (19)
N5—C25—H25	118.7	N1—Cu1—N3	114.72 (18)
C24—C25—H25	118.7	N7—Cu1—N3	107.46 (19)
N6—C26—C27	122.8 (5)	N1—Cu1—N5	107.38 (19)
N6—C26—H26	118.6	N7—Cu1—N5	103.62 (19)
C27—C26—H26	118.6	N3—Cu1—N5	100.09 (19)
C26—C27—C28	120.1 (5)	N2—Cu2—N4^v^	118.26 (19)
C26—C27—H27	120.0	N2—Cu2—N8	114.40 (19)
C28—C27—H27	120.0	N4^v^—Cu2—N8	103.2 (2)
C27—C28—C29	116.0 (6)	N2—Cu2—N6^vi^	111.83 (19)
C27—C28—C23	121.5 (5)	N4^v^—Cu2—N6^vi^	103.47 (19)
C29—C28—C23	122.5 (5)	N8—Cu2—N6^vi^	104.15 (19)

Symmetry codes: (i) −*x*+1, −*y*, −*z*+1; (ii) −*x*−3, −*y*, −*z*+2; (iii) *x*+2, −*y*+1/2, *z*−1/2; (iv) *x*, *y*, *z*−1; (v) *x*−2, −*y*+1/2, *z*+1/2; (vi) *x*, *y*, *z*+1.
